# Supplementary material for: The interactome of intact mitochondria by cross-linking mass spectrometry provides evidence for coexisting respiratory supercomplexes
Source: Mol Cell Proteomics. 2017 Dec 8;17(2):216–32. doi: 10.1074/mcp.RA117.000470 (PMC5795388; doi:10.1074/mcp.RA117.000470)
Supplement: Supplemental Data [file supp_RA117.000470_133922_0_supp_23978_fzffwf.zip › spectra_annotation/mito_spectra_annotation/183-1-5-1-7-1.pdf]

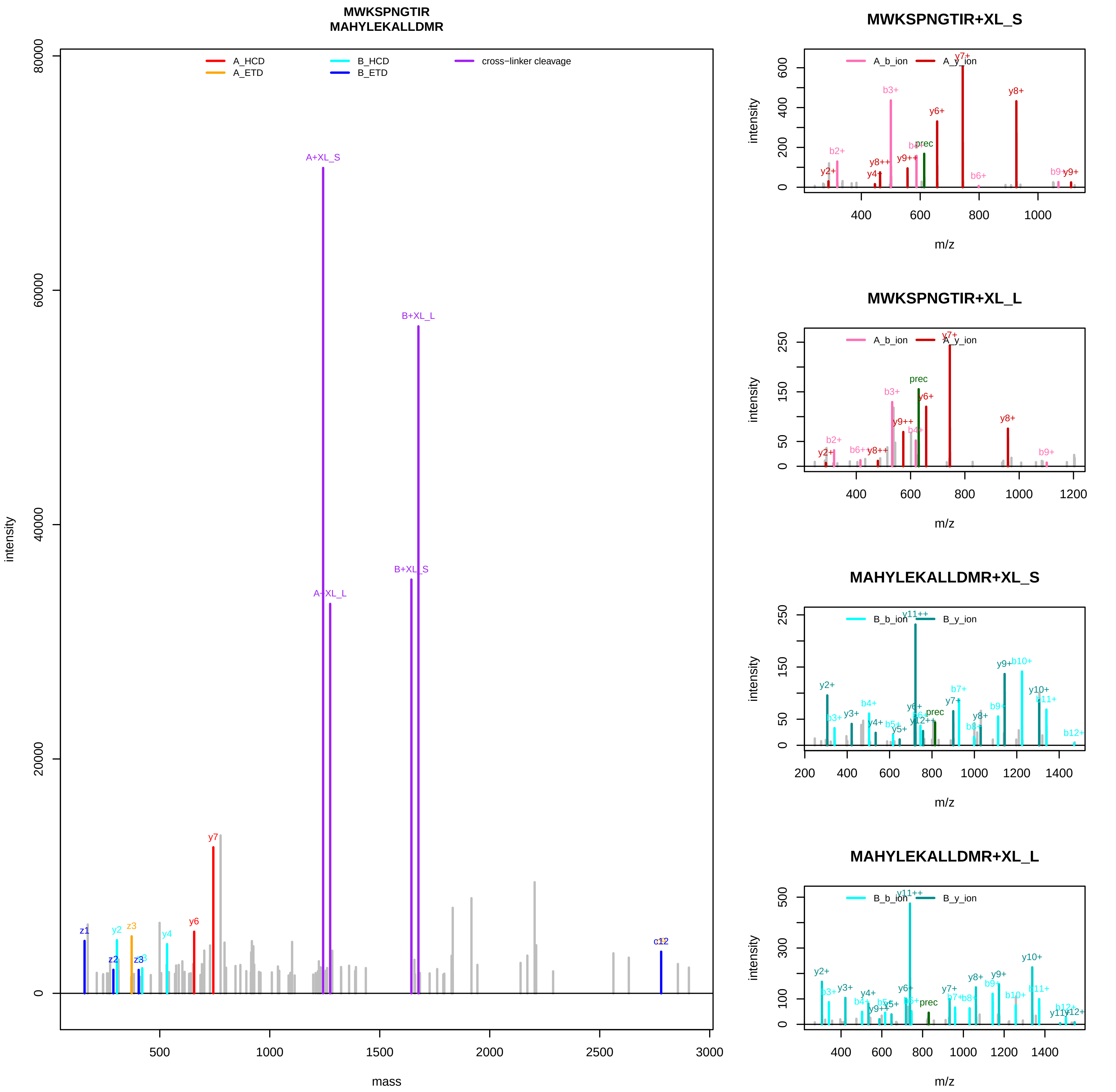

MWKSPNGTIR
MAHYLEKALLDMR
A_HCD
A_ETD
B_HCD
B_ETD
cross−linker cleavage
A+XL_S
B+XL_L
B+XL_S
A+XL_L
y7
y6
y4
y2
z1
z2
z3
c12
intensity
mass
0
20000
40000
60000
80000
500
1000
1500
2000
2500
3000
MWKSPNGTIR+XL_S
A_b_ion
A_y_ion
y7+
b3+
y8+
y6+
prec
b2+
y8++
y9++
y4+
y2+
b6+
b9+
y9+
intensity
m/z
MWKSPNGTIR+XL_L
A_b_ion
A_y_ion
prec
b3+
y6+
y9++
y8+
b2+
b6+
y8++
y2+
b9+
intensity
m/z
MAHYLEKALLDMR+XL_S
B_b_ion
B_y_ion
y11++
y9+
b10+
y2+
b7+
y10+
b11+
y7+
b4+
y3+
b3+
y4+
prec
y8+
b9+
y5+
b12+
intensity
m/z
MAHYLEKALLDMR+XL_L
B_b_ion
B_y_ion
y11++
y10+
y2+
y8+
y9+
b9+
b11+
y3+
y4+
b3+
y7+
b7+
b8+
b10+
b4+
b5+
y5+
y6+
prec
b12+
y12+
intensity
m/z
